# Multimodal deep learning for objective skill assessment in robot-assisted vesico-urethral anastomosis

**DOI:** 10.1007/s11701-026-03290-z

**Published:** 2026-03-10

**Authors:** Somayeh B. Shafiei, Saeed Shadpour, Anthony Dakwar, Zhaomin Xu, James L. Mohler

**Affiliations:** 1 Intelligent Cancer Care Laboratory, Department of Urology, Roswell Park Comprehensive Cancer Center, Buffalo, NY 14263 USA; 2 Department of Animal Biosciences, University of Guelph, Guelph, ON N1G 2W1 Canada; 3 Department of Surgical Oncology, Roswell Park Comprehensive Cancer Center, Buffalo, NY 14263 USA; 4 Department of Surgery, Colorectal Surgery, University of Rochester Medical Center (URMC), Rochester, NY 14642 USA; 5 Department of Urology and Lineberger Comprehensive Cancer Center, University of North Carolina at Chapel Hill, Chapel Hill, NC 27514 USA

**Keywords:** Vesico-urethral anastomosis (VUA), Surgical skill assessment, Competency evaluation, Multimodal deep learning

## Abstract

This study aimed to classify robot-assisted surgery (RAS) skill levels (inexperienced, competent, and experienced) during performance of vesico-urethral anastomosis (VUA) using multimodal physiological signals. We trained Convolutional Neural Networks (CNNs) and Long Short-Term Memory (LSTM) models on data collected from 23 RAS (RAS trainees and experienced surgeons) performing two VUAs on animal tissue. The dataset included 116-channel electroencephalogram (EEG) and 20 eye-tracking signals recorded during two VUA subtasks: (1) needle grasping, positioning, and entry; and (2) needle driving with wrist rotation and suture pull-out. Skill levels were rated by three raters using the Robotic Anastomosis Competency Evaluation (RACE) tool. Hyperparameters of the models were tuned using grid search with group 4-fold cross-validation on 16 participants and final model performance was evaluated on data from 7 unseen (held-out test) participants, repeated over 10 iterations. Weighted F-scores for classifying skill level using EEG and eye-tracking data were 0.84 for subtask 1 and 0.89 for subtask 2. Using paired t-tests, high-density EEG (116 channels) significantly outperformed low-density EEG (32 channels) for subtask 1 (*p* = 0.001), with no difference for subtask 2 (*p* = 0.15). Adding eye-tracking data significantly improved classification for subtask 2 (*p* = 0.001), but not for subtask 1 (*p* = 0.5). Multimodal deep learning using EEG and eye-tracking data enabled objective classification of surgical skills during VUA. The benefits of high-density EEG and multimodal integration were task-dependent, underscoring the need to tailor assessment tools to the cognitive and sensorimotor demands of specific surgical subtasks.

**Supplementary Information:**

The online version contains supplementary material available at 10.1007/s11701-026-03290-z.

## Introduction

### Challenges in RAS skill assessment and existing approaches

Robot-assisted surgery (RAS) has been widely adopted across multiple surgical specialties and has transformed surgical practice by enabling enhanced visualization, improved precision, and reduced blood loss, with potential benefits for clinical outcomes. A critical component of robot-assisted radical prostatectomy (RARP) is vesico-urethral anastomosis (VUA). However, assessing and acquiring this skill remains challenging due to task complexity, subjective evaluations, and the absence of efficient surgical performance assessment tools.

Manual evaluation tools such as the Global Evaluative Assessment of Robotic Skills (GEARS) [1] and Robotic Anastomosis Competency Evaluation (RACE) [2] rely on human scorers and, although foundational, are time-intensive. Automated performance metrics (APMs) have been explored to address these limitations. For instance, Hung et al. used kinematic data from the da Vinci Research Kit to predict surgical skill and showed promising results [3]. Nonetheless, kinematic metrics fail to capture the cognitive skills that are critical to surgical expertise [4].

Recently, attention has shifted toward physiological signals for expertise assessment. Electroencephalogram (EEG) and eye-tracking data have shown promise as markers of cognitive load and visual attention. Ahmidi et al. demonstrated how gaze patterns could reflect RAS skill levels [5]. Also, EEG has been used to distinguish between novice and expert performance on surgical tasks [6]. However, these studies typically analyzed each modality separately, leaving a gap in understanding the value of multimodal data integration.

EEG offers high temporal resolution and provides detailed information about neural activity during surgical tasks, including attention, workload, and engagement, factors essential for skill development and efficiency [7, 8]. EEG also captures cortical responses associated with learning and neuroplasticity, which provide valuable information for tracking training progress and cognitive load during surgery [9–11].

### Roles of machine learning and deep learning in surgical skill assessment

Machine learning and deep learning methods are increasingly applied in surgical skill assessment due to their capacity to process high-dimensional, multimodal data [12]. EEG and eye-tracking data, which reflect cognitive and visual processes, are well-suited for such models [9, 11].

Deep learning architectures such as Convolutional Neural Networks (CNNs) and Long Short-Term Memory (LSTM) networks have achieved notable success in sequence analysis tasks [13]. CNNs extract spatial features, while LSTMs model temporal dependencies. Their combination in CNN-LSTM architecture enables spatiotemporal pattern recognition in complex physiological data. Addition of an attention mechanism allows models to focus on the most informative input regions to improve classification accuracy [14, 15].

### Current study’s focus

Prior studies have advanced expertise prediction using various data types and models, but few have integrated multiple physiological and behavioral signals to assess skill in Robot-Assisted Vesico-Urethral Anastomosis subtasks. This study addresses that gap by developing a deep learning model that fuses EEG and eye-tracking data to predict expertise in VUA subtasks.

We collected EEG and eye-tracking data from participants with varying experience levels during performance of VUA tasks. The proposed model aims to offer a robust, objective framework for surgical skill evaluation by using these physiological and behavioral signals and deep learning techniques. This model could potentially support customized training programs by identifying specific areas for improvement and assisting with objective performance evaluation.

## Materials and methods

This study was approved by the Institutional Review Board at Roswell Park Comprehensive Cancer Center (IRB Protocol I-241913). Participants were recruited via email, in-person invitations, and flyers. The IRB waived the requirement for written consent; participants received an information sheet and provided verbal consent.

### Dataset

Twenty-three right-handed participants (mean age: 35 ± 12 years; 16 males, 7 females) took part in this study including nine pre-medical students, two scientists, three residents, four fellows, and five surgeons who had varying levels of RAS experience. A heterogeneous participant pool ranging from pre-medical students to experienced surgeons was intentionally included to capture a broad spectrum of robotic surgery expertise. This diversity ensured sufficient representation of skill levels (from inexperienced to highly experienced), necessary for developing and validating a robust skill classification model capable of generalizing across different experience levels.

**Fig. 1:**
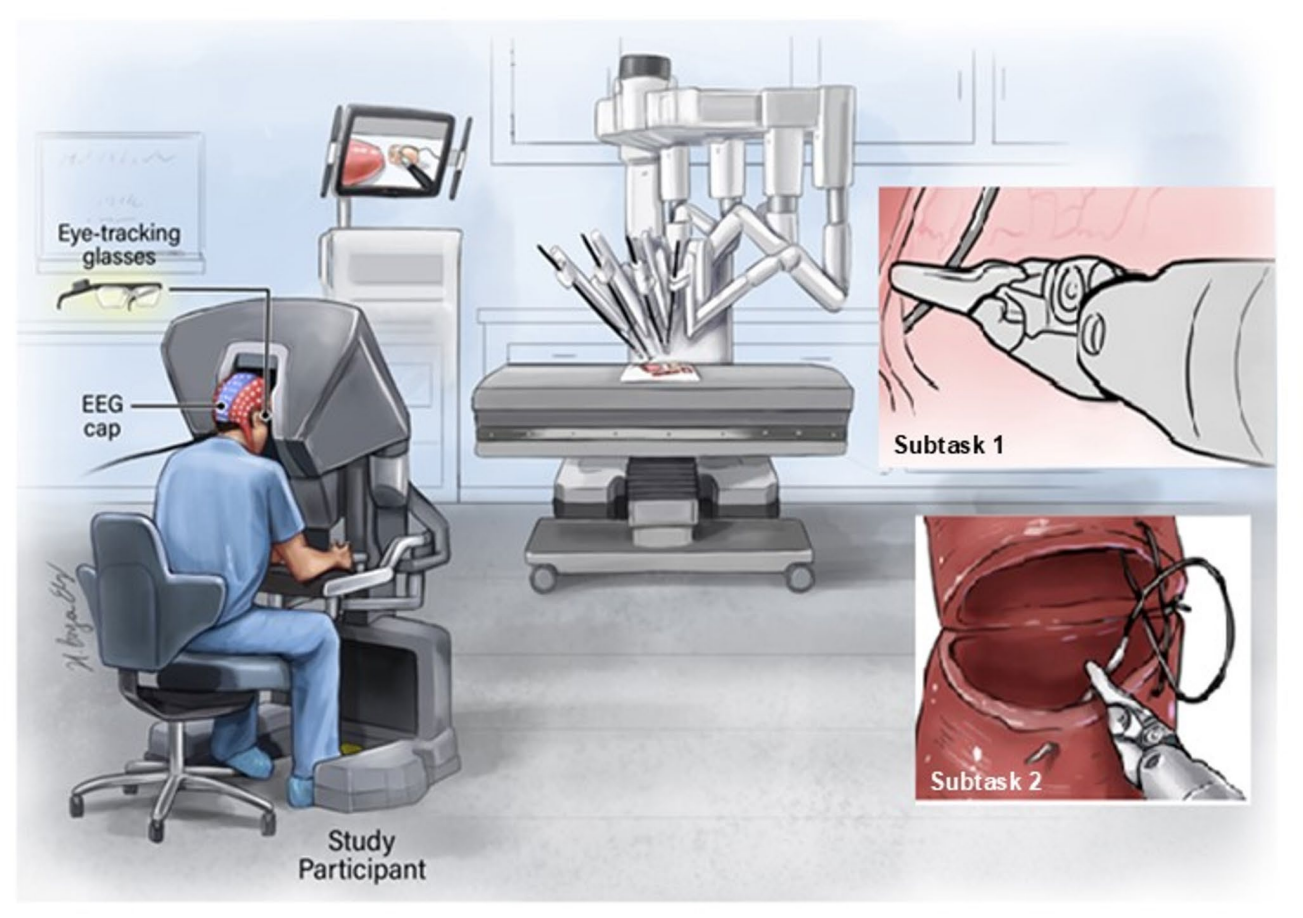
Representation of experimental setup. EEG and eye-tracking data were collected while participants used the da Vinci surgical robot to perform anastomoses subtasks on animal tissue

Each participant performed two anastomoses using animal tissue (porcine esophagus or intestine; Fig. 1). VUA was selected as the representative task for this study because it constitutes one of the most technically demanding and clinically consequential steps of RARP. Successful completion of VUA requires precise bimanual coordination, strong depth perception, and sustained cognitive engagement. This makes it an ideal setting for evaluating multimodal physiological markers of expertise. Moreover, performance in VUA varies greatly across different experience levels, from trainees to expert surgeons. This makes it well suited for developing and validating skill classification models. The availability of the validated RACE tool further provides a framework for ground-truth labeling of performance, which facilitates rigorous model training and evaluation under realistic surgical conditions.

Simultaneous and synchronized EEG (512 Hz) and eye-tracking (50 Hz) data were recorded. A synchronization box from AntNeuro^®^ was used to deliver digital triggers to both systems to align events, such as stimulus onset, for accurate data integration. Physiological signals such as EEG and eye-tracking provide quantitative insight into cognitive and sensorimotor processes that underlie surgical skill. These neurobehavioral states are associated with task precision, error rates, and operative efficiency, all of which have been linked to surgical outcomes such as tissue trauma and postoperative recovery [5, 6, 16].

Video recordings of each anastomosis were reviewed to identify the start and end of two predefined subtasks based on standardized operational definitions. **Subtask 1** began when the needle was first grasped and positioned for tissue entry and ended upon successful needle entry. **Subtask 2** began with needle driving through tissue and wrist rotation and ended upon completion of suture pull-out and positioning over the posterior plate. Segmentation was performed using visual cues in the recorded videos and synchronized system timestamps to ensure accurate alignment with EEG and eye-tracking data.

### Ground-truth skill levels in performing RAS anastomosis subtasks

Three raters (two RAS surgeons and one trained scientist) independently scored each segmented subtask using the RACE Likert-scale criteria (Supplement 1), a validated tool designed to evaluate RAS anastomosis performance across six domains [2, 17]: (1) needle positioning; (2) needle entry; (3) needle driving and tissue trauma; (4) suture placement; (5) tissue approximation; and (6) knot tying. Each domain is scored on a 5-point Likert scale. Raters were blinded to participant identity, training background, and experience level, and evaluated performance solely based on the de-identified video recordings.

RACE scores for needle positioning and needle entry were used to assess performance on Subtask (1). Scores for needle driving and tissue trauma, suture placement, and tissue approximation were used for Subtask (2). Knot tying samples (*n* = 62) were insufficient to support stable training and generalization of the deep learning model in this multi-class setting and therefore were excluded from analysis.

Participants were classified into three skill levels based on RACE scores [2]: Class 1 (inexperienced, poor performance); Class 2 (competent, intermediate performance); Class 3 (experienced, ideal performance). 

Inter-rater agreement for raw RACE Likert-scale scores was assessed using the intraclass correlation coefficient (ICC; two-way random-effects model, absolute agreement) that yielded ICCs of 0.60 (95% CI: 0.54–0.81), 0.65 (0.50–0.76), 0.71 (0.52–0.84), 0.76 (0.65–0.87), and 0.72 (0.53–0.85) for RACE domains 1–5, respectively.

Inter-rater reliability among the raters’ categorical skill assessments was evaluated using Fleiss’ Kappa (κ). For categorical skill labels, disagreements were resolved using majority voting across raters. According to the widely accepted interpretation by Landis and Koch, κ values below 0.00 indicate poor agreement, 0.00–0.20 slight, 0.21–0.40 fair, 0.41–0.60 moderate, 0.61–0.80 substantial, and 0.81–1.00 almost perfect agreement [18]. The results showed a moderate level of agreement among raters for Subtask 1 (κ = 0.6, 95% CI 0.50–0.78) and substantial agreement for Subtask 2 (κ = 0.72, 95% CI 0.57–0.85).

### Sample size and class balance considerations

Although the number of participants (*n* = 23) may appear modest so subject-independent training and testing with repeated group-based cross-validation were applied, which is the recommended evaluation strategy for physiological time-series modeling to prevent data leakage and overestimation of performance.

Model training was performed on hundreds of temporally segmented samples per subtask (440 samples per subtask after preprocessing) derived via label-preserving sliding windows that provided sufficient data for learning task-relevant spatiotemporal patterns while maintaining strict subject-level independence.

Class imbalance arising from a higher number of inexperienced participants was addressed via random downsampling of the majority class to equalize class distributions; in total, 66 samples were removed prior to model development. Ultimately, 440 samples per subtask were used for model development and testing. Synthetic resampling can introduce non-physiological temporal and spectral artifacts in neural signals; therefore, downsampling was used to preserve biological signal structure. Downsampling was selected to avoid introducing synthetic signal structure, which may be problematic for neurophysiological data. Class weighting was applied during training to mitigate residual imbalance effects. This conservative strategy prioritizes generalizability and robustness over maximizing sample count.

### EEG and eye-tracking data pre-processing

Electrodes F8, POz, AF4, AF8, F6, FC3, M1, and M2 (placed on the mastoids) were excluded due to poor signal quality. The remaining 116 EEG channels were cleaned of artifacts using the Advanced Source Analysis (ASA) software (ANT Neuro Inspiring Technology Inc., Hengelo, the Netherlands), a comprehensive EEG analysis package that provides interactive review, filtering, and artifact correction tools.

- A 60 Hz notch filter was applied to remove line noise.
- The data were band‑pass filtered between 0.2 Hz and 250 Hz (24 dB/octave).
- Signals were re-referenced using the common average reference method [19].
- Continuous EEG data were inspected visually using ASA to identify segments exhibiting artifactual activity (e.g., ocular, muscular, or movement artifacts). Artifact components were removed using blind source separation and topographical Principal Component Analysis (PCA), as implemented in ASA [19].
- A spatial Laplacian transform was applied to the cleaned signals to reduce volume conduction effects [20, 21].

All preprocessing steps were applied identically across subjects to ensure consistency.

Eye-tracking data were processed using Tobii Pro Lab© software. A moving average filter with a window size of three points was applied to reduce noise. The exported dataset contained 20 time-series signals, which included:

1–2) Gaze point (X, Y).

3–5) Gaze point 3D (X, Y, Z).

6–8) Gaze direction (left: X, Y, Z).

9–11) Gaze direction (right: X, Y, Z).

12–14) Pupil position (left: X, Y, Z).

15–17) Pupil position (right: X, Y, Z).

18) Pupil diameter (left).

19) Pupil diameter (right).

20) Eye movement type index (e.g., 1: fixation, 2: saccade, 3: unclassified/eyes not detected).

Each data point was timestamped in milliseconds by the recording systems to enable precise synchronization between EEG and eye-tracking signals. A smoothed pairwise polynomial spline interpolation was applied to align sampling rates and ensure temporal matching across modalities.

### Model input preparation

Missing values were handled using mean imputation. Prior to model input, EEG and eye-tracking signals were standardized using channel-wise z-score normalization to reduce scale differences across modalities and improve training stability of the CNN–LSTM model.

We applied time-series-specific data augmentation techniques to expand the dataset and improve classification performance [22, 23]. A label-preserving cropping approach was applied using a sliding window with a 50% overlap before feeding data into the network to extract fixed-size motion subsequences from each trial. Each subsequence inherited the original trial’s class label to maintain label consistency. Sliding windows of 3, 5, and 10 s with 50% overlap were tested on both EEG and eye-tracking data to determine the optimal window size for maximizing CNN-LSTM model performance. Window length plays a crucial role: shorter windows may miss temporal context, while longer ones can weaken important temporal features.

Data were structured as (10, L × 51.2, 136) for model input, where L was the window length in seconds scaled by 51.2 to standardize sampling intervals, and 136 represented the feature count (116 EEG + 20 eye-tracking features). This format captured the temporal dynamics essential in sensor-based analysis. For trials shorter than 5–10 s, common among experienced participants, zero-padding was applied at the end of the signals to meet the required input length. An attention layer was integrated into the model to focus on informative regions of the input while ignoring padded segments to enhance classification accuracy.

Each sliding window (3, 5, or 10 s) was subdivided into T = 10 temporal steps, independent of window duration, to provide a consistent temporal structure for sequence modeling. Model inputs were represented as four-dimensional tensors of shape *(N*,* T*,* L*,* F)*, where *N* is the number of samples, *T = 10* is the number of temporal steps per window, *L* is the number of samples per step, and *F* is the number of input features. The choice of T = 10 temporal steps per window was made to provide a fixed-length temporal representation compatible with the LSTM architecture and preserve sufficient temporal resolution within each sliding window. This design balances temporal context with computational tractability and was validated empirically during preliminary experiments.

### CNN-LSTM classification model to predict skill level in performing anastomosis subtasks

The CNN-LSTM architecture leveraged the complementary strengths of both networks: CNNs effectively extract high-level spatial features from multichannel sensor data, while LSTMs model temporal dependencies across time-series inputs. This hybrid framework is well-suited for applications that require a detailed understanding of both spatial and temporal dynamics, such as classifying complex physiological signals like EEG and eye-tracking data.

**Fig. 2:**
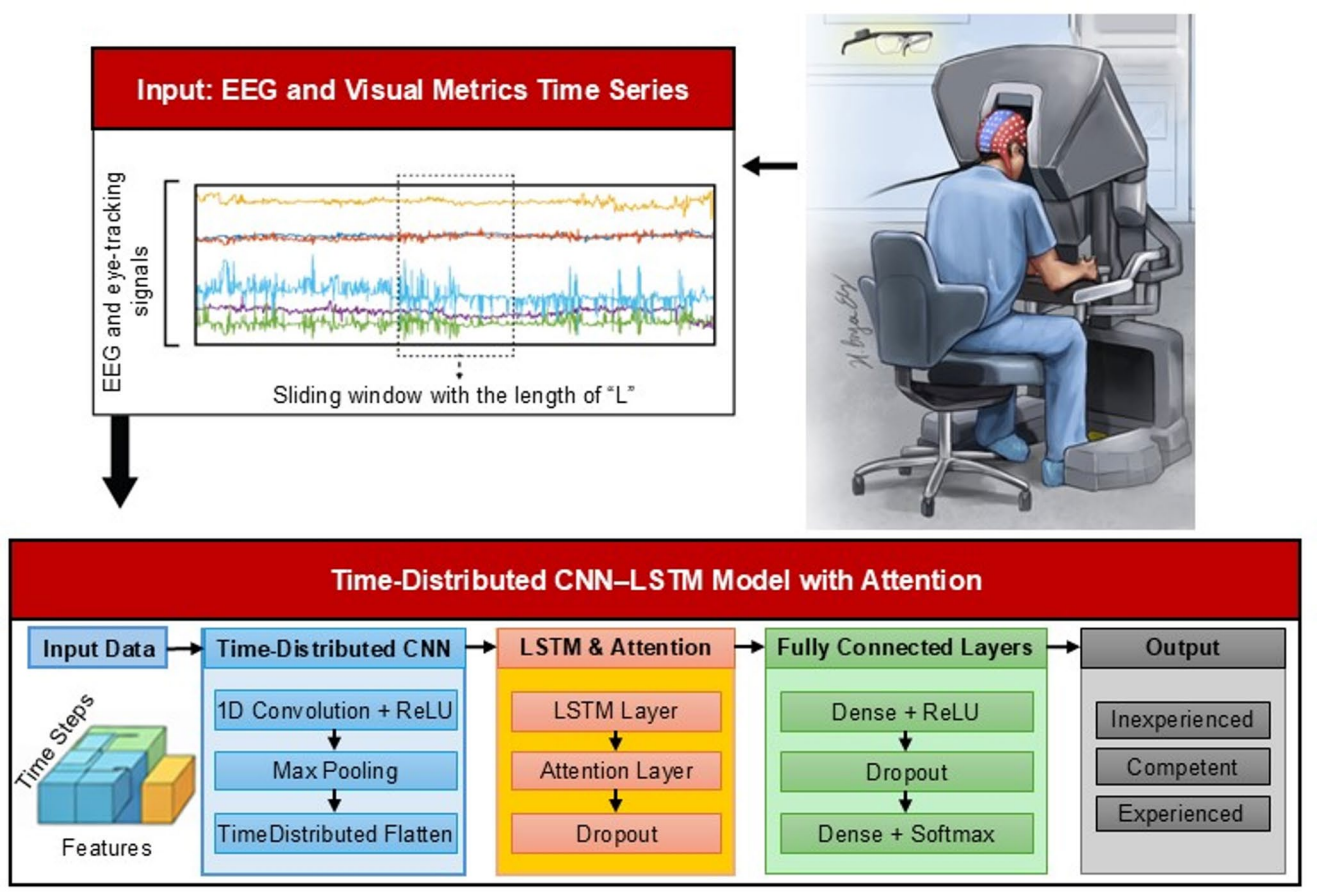
Overview of the CNN–LSTM model with attention for surgical skill classification. EEG and eye-tracking signals were segmented using label-preserving sliding windows with 50% overlap and input to a time-distributed CNN for feature extraction. Temporal dependencies were modeled using an LSTM layer followed by an attention mechanism and fully connected layers with dropout regularization. The model outputs skill level classifications (inexperienced, competent, experienced)

We framed the surgical skill level evaluation as a supervised three-class classification task. Inputs to the model were multivariate time-series from EEG and eye-tracking signals (Fig. 2). The model output was the predicted skill level:

- Class 1: Inexperienced.
- Class 2: Competent.
- Class 3: Experienced.

Ground-truth labels were derived from expert assessments using the RACE metrics. The CNN-LSTM model was trained using categorical cross-entropy loss to optimize predicted class probabilities over the three levels.

#### Model architecture

- *Convolutional Layer*: A 1D convolutional layer was applied to each timestep independently using a TimeDistributed wrapper in Keras. This layer used 64 filters, kernel size 5, Rectified Linear Unit (ReLU) activation, and L2 regularization to reduce overfitting.
- *Max Pooling*: A max pooling layer followed the convolution layer to reduce feature dimensionality, lower computational cost, and minimize overfitting risks.
- *Flattening*: Output from the convolutional layers was flattened into a single vector per timestep to feed it into the LSTM layer.
- *LSTM Layer*: The LSTM layer captured temporal dependencies across the sequence. The L2 regularization was applied for this layer to control overfitting.
- *Attention Layer*: The attention mechanism layer was positioned after the LSTM to enable the model to focus on the most informative parts of the signal, particularly beneficial for variable-length inputs and padded sequences.
- *Dropout Layers*: Dropout was applied after the attention and dense layers to prevent overfitting by randomly deactivating a subset of neurons during training.
- *Output Layers*: The network concluded with a fully connected dense layer with ReLU activation, followed by a final dense layer using Softmax activation to output class probabilities for the three skill levels.

#### Training and validation

Hyperparameter tuning was performed using grid search with group-based 4-fold cross-validation on data from 16 randomly selected participants. Within each fold, models were trained on samples from a subset of participants and validated on samples from held-out participants to ensure strict subject-level separation between training and validation sets. GroupKFold was used to prevent data leakage by ensuring that data from the same participant were never present in both training and validation folds. Model selection was based on mean cross-validation performance across folds. Final model performance was evaluated on data from 7 unseen participants. The participant-level split into 16 training/validation participants and 7 held-out test participants repeated 10 times using different random partitions to assess robustness and generalizability.

We tested a variety of hyperparameters to optimize the model’s performance that included:

- LSTM Units: Different configurations with 50, 100, 150, and 200 units were tested to determine the optimal balance between model complexity and processing efficiency.
- Convolutional Filters: Filters in the configurations of 32, 64, and 128 were tested to fine-tune the feature extraction capabilities of the network.
- Kernel Size: Investigated kernel sizes of 3 and 5 to optimize spatial feature aggregation.
- Dropout Rate: Explored rates of 0.5, 0.6, and 0.8 to prevent overfitting by reducing co-dependence among neurons.
- L2 Regularization: Tested values of 0.1, 0.01, and 0.001 to control the model’s complexity and enhance its generalization capabilities.
- Epochs: Varied the training durations with 50 and 100 epochs to determine the sufficient number of iterations for the network to converge effectively; and
- Batch Size: Examined sizes of 64, 128, and 256 to optimize the computational load and model training dynamics.

Grid search with Cross-Validation was used to identify optimal hyperparameters by training multiple models on different subsets of the data. Early stopping was used to prevent overfitting by stopping training if the loss did not improve for 10 consecutive epochs.

Class weights were computed using the compute_class_weight function from the scikit-learn library to mitigate the effects of class imbalance. This method assigns weights inversely proportional to class frequencies in the training dataset to ensure that underrepresented classes were assigned higher weights to contribute more strongly to the loss function. The resulting class weights were incorporated into model training by weighting the categorical cross-entropy loss to reduce bias toward majority classes.

Samples from the remaining 7 participants were used to test the performance of the developed model. Model development and testing were repeated 10 times. The actual and predicted skill levels for test samples across all repetitions were used to calculate the model’s performance metrics.

CNN-LSTM models were developed in Python 3.7 using TensorFlow (2.9.1). Models were developed using 32-channel EEG data, 116-channel EEG data, eye-tracking data, and their combination. Performance was compared using paired t-tests.

### Model’s performance evaluation metrics

True positives (TP) referred to instances where the model accurately identified the positive class. False positives (FP) denoted cases where the model incorrectly labeled a sample as positive when it was actually negative. The performance of the developed models for classifying the surgical skill levels of participants was evaluated using various measurements that included:

- Confusion matrices were used to evaluate the performance of the classification models by comparing the actual and predicted labels.
- Average accuracy: The proportion of correctly classified samples relative to the total number of samples; and.
- The weighted F1-score was computed to evaluate overall classification performance across all classes while accounting for class imbalance. First, the confusion matrix was used to derive per-class precision and recall, defined respectively as:
- Precision: The ratio of TP to all predicted positives (TP + FP).
- Recall: The ratio of TP to all actual positives (TP + FN).

The per-class F1-score was calculated as the harmonic mean of precision and recall:

$$\:F1-score=\frac{2\times\:(recall\times\:precision)}{recall+precision}$$

The weighted F1-score was computed as a weighted average of the per-class F1-scores, where the weights correspond to the number of true instances (support) for each class:

$$\:Weighted\:F1-score=\frac{\sum_{i}\:{F1}_{i}\times\:{Support}_{i}}{\sum_{i}\:{Support}_{i}}$$

This process ensured that classes with more samples contributed proportionally to the final metric, making the overall performance reflective of the dataset’s class distribution in the presence of class imbalance. 

### Statistical analysis

Model performance was evaluated using repeated independent runs to account for stochastic variation in training and initialization. Models were trained and evaluated 10 times for each modality and EEG configuration and these repeated runs constituted the unit of analysis. Two-tailed paired-sample t-tests were used to compare performance metrics between modalities and EEG configurations; pairing was defined across corresponding runs. Normality of paired differences was not tested formally due to the small sample size, but paired t-tests were deemed appropriate given their robustness to moderate deviations from normality and the controlled experimental setting. Holm–Bonferroni correction was applied to control for multiple comparisons and corrected p-values are reported.

## Results

**Fig. 3:**
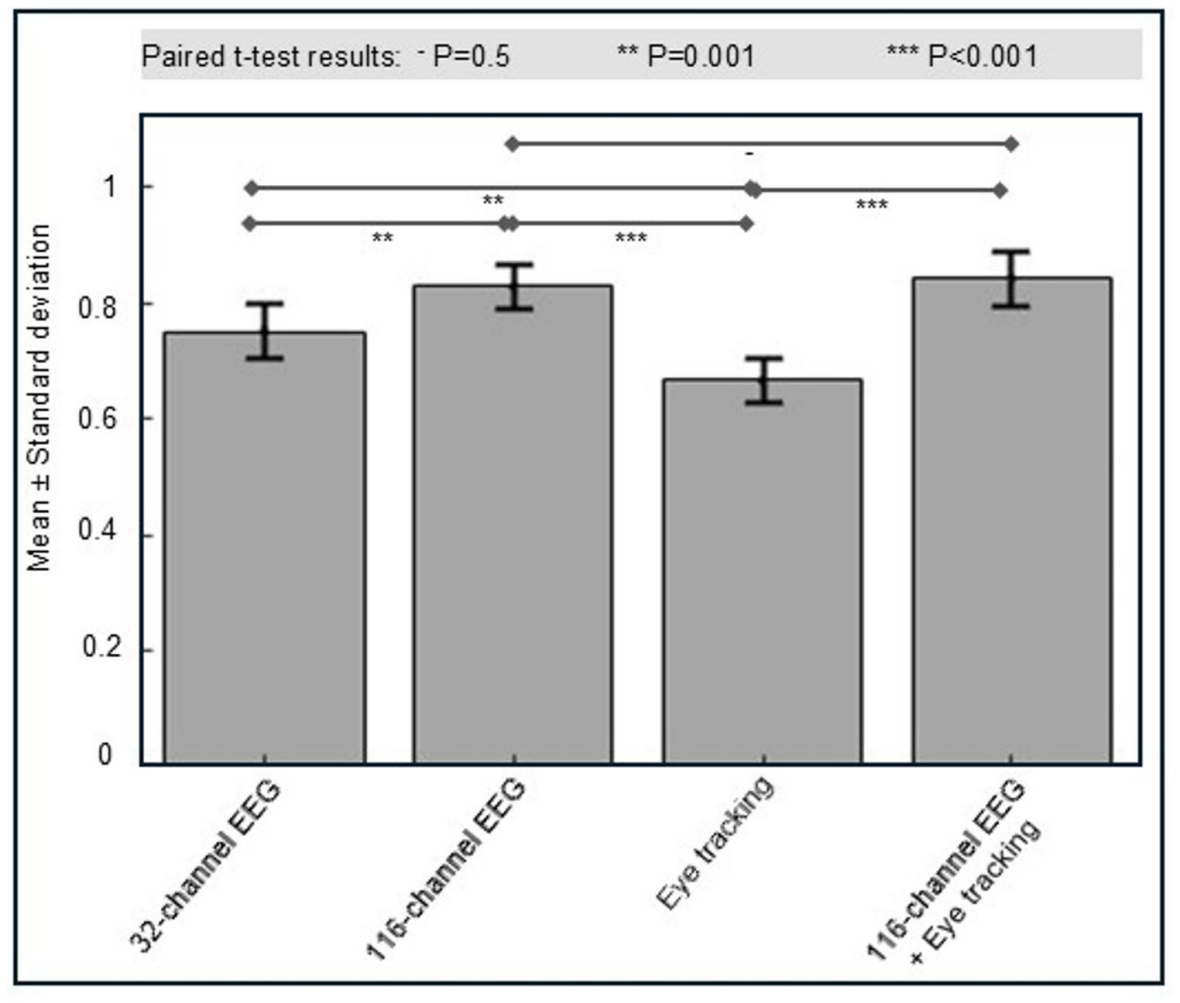
Comparison of weighted F1-scores for CNN–LSTM classification models trained using different data modalities for Subtask 1. The x-axis denoted the input modality (32-channel EEG, 116-channel EEG, eye tracking, and combined 116-channel EEG + eye tracking), and the y-axis represented the weighted F1-score. Bars indicated mean values, and error bars represented standard deviation. Statistical significance between modality pairs was assessed using paired t-tests; “–” denoted non-significant differences (*p* = 0.5), “**” denoted *p* = 0.001, and “***” denoted *p* < 0.001

**Fig. 4:**
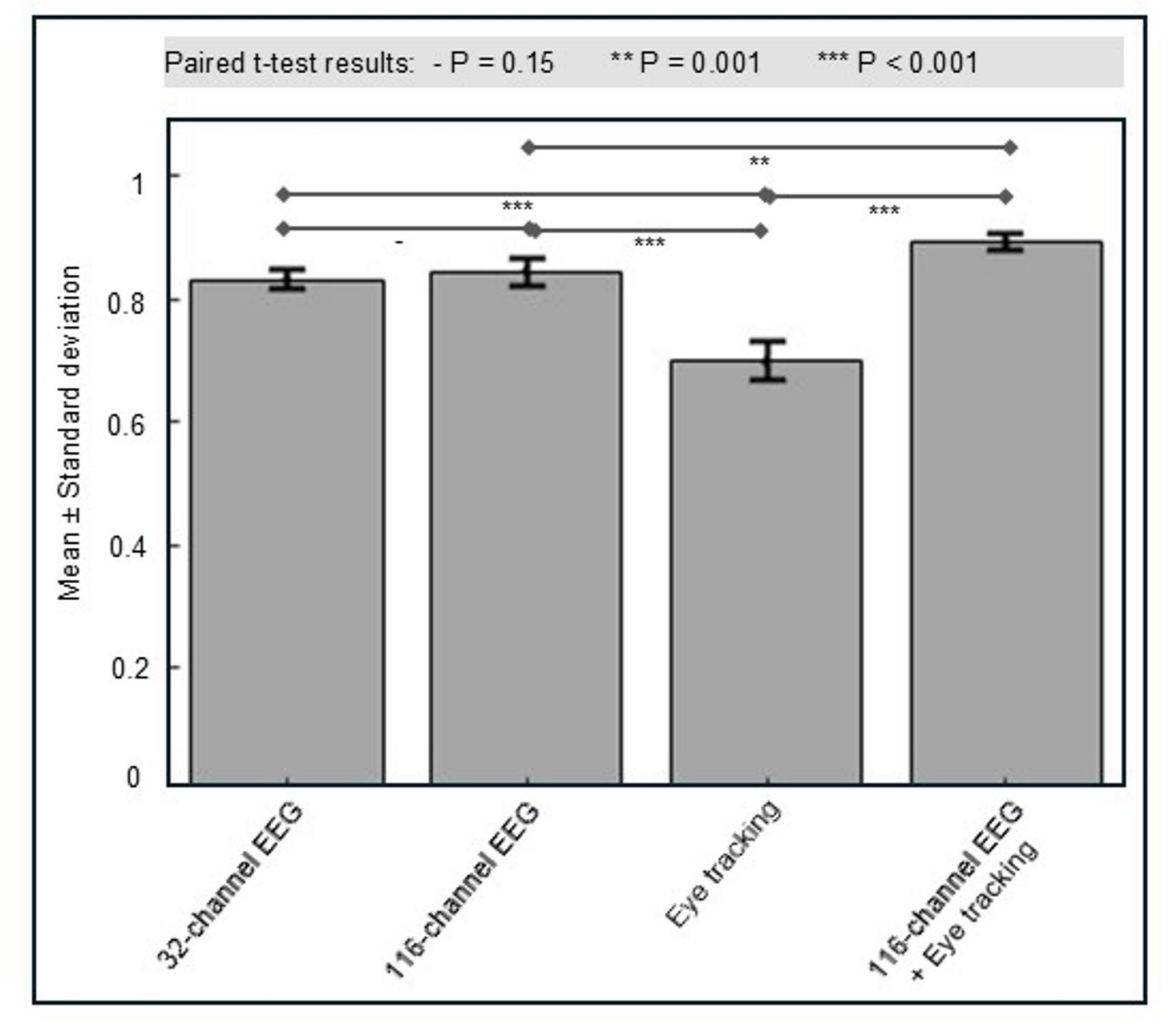
Comparison of weighted F1-scores for CNN–LSTM classification models trained using different data modalities for Subtask 2. The x-axis denoted the input modality (32-channel EEG, 116-channel EEG, eye tracking, and combined 116-channel EEG + eye tracking), and the y-axis represented the weighted F1-score. Bars indicated mean values, and error bars represented standard deviation. Statistical significance between modality pairs was assessed using paired t-tests; “–” denoted non-significant differences (*p* = 0.15), “**” denoted *p* = 0.001, and “***” denoted *p* < 0.001

Sliding window lengths of 3, 5, and 10 s were tested. The 5-second window was selected because it achieved the best overall classification performance. The 3-second window resulted in degenerate behavior, with the model collapsing to a single-class prediction. Accuracy appeared high for one class but performance was near-zero for the remaining classes. Hence, summary metrics such as accuracy or F1-score were not representative of true multi-class performance. This outcome indicates that a 3-second window does not capture sufficient temporal context for skill discrimination. The 10-second window did not yield further performance improvements over the 5-second window. Skill-level classification results for subtasks 1 and 2 were shown in Figs. 3 and 4, respectively.

Table 1 presented the performance metrics of the developed CNN-LSTM models for classifying skill level in performing anastomosis subtasks.

**Table 1:** Performance of CNN-LSTM models developed for predicting skill levels in anastomosis subtasks 1 and 2

Data modalities	Subtask 1			
	A	B	C	D
Precision	0.76 ± 0.04	0.82 ± 0.04	0.68 ± 0.05	0.85 ± 0.04
Recall	0.77 ± 0.02	0.81 ± 0.03	0.68 ± 0.04	0.84 ± 0.05
Accuracy	0.75 ± 0.04	0.83 ± 0.02	0.66 ± 0.04	0.84 ± 0.05

Data modalities	Subtask 2			
	A	B	C	D
Precision	0.83 ± 0.2	0.84 ± 0.01	0.73 ± 0.03	0.89 ± 0.01
Recall	0.82 ± 0.02	0.81 ± 0.02	0.71 ± 0.03	0.89 ± 0.01
Accuracy	0.83 ± 0.01	0.84 ± 0.02	0.70 ± 0.03	0.89 ± 0.01

The results represent the outcomes from 10 repeated predictions performed on unseen test samples from 7 randomly selected participants. The input modalities were as follows: A: 32-channel EEG, B: 116-channel EEG, C: Eye tracking, D: 116-channel EEG + Eye tracking

**Fig. 5:**
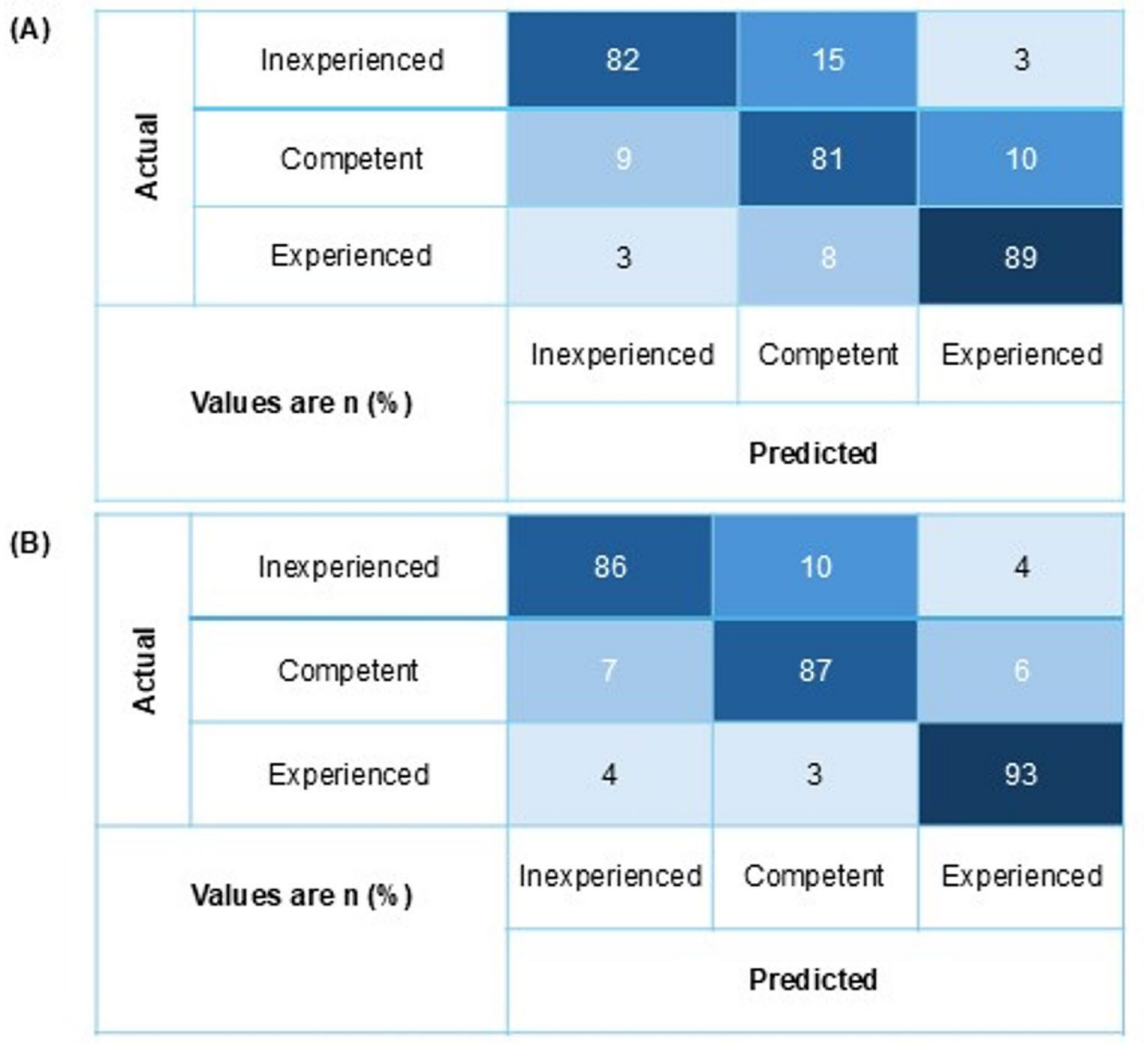
Confusion matrices for skill-level prediction using the CNN–LSTM model for Subtask 1 (**A**) and Subtask 2 (**B**). Rows corresponded to actual skill labels and columns corresponded to predicted labels. Values were reported as percentages of samples within each true class. The model was evaluated on unseen samples from 7 randomly selected participants across 10 replicates using combined 116-channel EEG and eye-tracking data. Skill level classes were defined as inexperienced, competent, and experienced

Confusion matrices for Subtask 1 and Subtask 2 using eye-tracking and 116-channel EEG data are presented in Fig. 5. The skill levels were categorized as inexperienced, competent, or experienced. The diagonal elements of the matrix indicated correct classifications.

## Discussion

The adoption of RAS continues to expand rapidly across surgical specialties to extend minimally invasive approaches for increasingly complex procedures and contribute to improved postoperative outcomes. Recent population-level analyses demonstrate that robotic platforms have enhanced clinical results in areas such as colorectal surgery, where statewide data show reduced complication and conversion rates compared to conventional approaches [24]. Similar benefits have been reported in thoracic surgery, with evidence of lower postoperative morbidity and shorter length of stay in robotic cases relative to open and video-assisted procedures [25]. Despite these advances, variability in surgeon performance remains a critical determinant of patient safety, and adverse events across specialties highlight persistent gaps in training and assessment. The need for robust, scalable, and objective methods to evaluate and improve technical proficiency becomes increasingly urgent as RAS continues to expand. In this context, our study demonstrated how multimodal signals, such as EEG and eye tracking, can provide reliable, data-driven insights into surgeon skill while performing complex tasks, such as VUA, to offer a foundation for next-generation training and competency assessment frameworks.

### Skill assessment in robotic surgery

Traditional training relies on subjective assessments that are prone to bias and variability. We proposed a data-driven deep learning framework that integrates EEG and eye-tracking data using a CNN-LSTM model to classify surgical expertise objectively.

Previous studies have focused on benchtop simulations using kinematic data, such as the JIGSAWS dataset [26–28] that use short, segmented gestures (suturing, needle-passing, knot-tying) performed on plastic models under highly controlled conditions. For example, deep CNNs achieved classification accuracies of 92.5%, 95.4%, and 91.3% for suturing, needle-passing, and knot-tying, respectively [27], and Nguyen et al.’s CNN-LSTM model achieved 98.4%, 98.4%, and 94.7% accuracy for the same tasks [28].

These studies report skill assessment accuracies exceeding 90%, but these values should not be compared to the present results due to substantial differences in task complexity and experimental setting. Prior work focused on isolated, gesture-level tasks on plastic phantom models under controlled benchtop conditions, whereas the current study evaluated performance during VUA on animal tissue, which involves continuous multi-step surgical execution, tissue deformation, variable anatomy, and higher cognitive and motor demands.

Differences in model training and evaluation approaches may contribute to discrepancies in reported performance. In many prior studies, models were evaluated on data from participants included in training and validation. The present study assessed generalization using unseen participants that provide a more rigorous and clinically relevant evaluation setting.

EEG and eye tracking are gaining attention for skill assessment. EEG features have differentiated expertise levels in laparoscopic simulations showing distinct spatial patterns between novices and experts [29]. Similarly, eye-tracking metrics, like fixation duration and gaze entropy, correlated with surgical experience [30]. Our prior work showed the value of EEG and eye-tracking engineered features for skill assessment across multiple platforms that included simulators, plastic models, and live pigs, and the suitability of machine learning for analyzing these signals [9–11, 31, 32].

The current study combined the EEG and eye-tracking data in a deep learning framework that demonstrated superior classification performance compared to single modality for subtask 2. High-density EEG (116 channels) further enhanced performance for one subtask that supports the findings of other studies that spatial EEG resolution improves decoding of complex cognitive and motor processes, particularly for cognitively demanding tasks [7, 8, 33, 34].

Our model applied a multimodal CNN–LSTM framework to RAS anastomosis subtasks using time-series EEG and eye-tracking data that enabled detailed, objective skill assessment beyond binary or subjective metrics.

### Effect of multimodal data in RAS skill assessment

Eye tracking alone yielded lower performance, particularly in Subtask 2, suggesting it was less effective for detecting skill differences under high cognitive load. In contrast, EEG, especially high-density EEG, showed higher classification accuracy. Combining eye tracking with 116-channel EEG significantly improved accuracy for Subtask 2 but not for Subtask 1.

These results reflect subtask-specific demands. Subtask 2 (needle driving with wrist rotation and suture pull-out) involved continuous motion, trajectory planning, and real-time adjustments, which required both motor planning (reflected in EEG) and visual feedback processing (reflected in eye tracking). Subtask 1 (needle grasping, positioning, and entry) consists of shorter, goal-directed actions that may rely less on continuous visual input, so adding eye tracking provided little additional information. The findings indicated that multimodal integration is most beneficial for subtasks that require sustained visual-motor coordination.

### Optimal EEG configuration for skill assessment

A notable finding was the difference between 32- and 116-channel EEG configurations. For Subtask 1, 116-channel EEG performed significantly better (*p* < 0.001), likely due to broader cortical coverage of regions involved in attention and motor planning. For Subtask 2, no significant difference was observed (*p* = 0.15).

These results suggest that high-density EEG provides advantages when tasks depend on localized neural activity and precise motor planning, as in Subtask 1. In contrast, Subtask 2 involves more continuous, distributed motor control that can be captured sufficiently by lower-density EEG. Overall, the benefit of high-density EEG appears task-dependent, with greater value for subtasks that require fine cognitive and motor control.

### Impact and implications of the findings

Our approach offers an objective RAS skill evaluation method by integrating EEG and eye tracking with deep learning. Unlike subjective assessments, this method delivers objective assessment that reduces variability in feedback.

Theoretically, this study positions physiological signals as reliable markers of expertise that lay the groundwork for data-driven surgical training systems that enhance learning efficiency and consistency. Practically, deploying these models in surgical training settings could potentially provide targeted feedback and support more individualized training pathways.

Although this study focused on physiological correlates of surgical skill rather than direct patient outcomes, the identified EEG and eye-tracking markers are linked to neurocognitive processes that underly technical performance. For example, stable frontal theta and reduced occipital alpha activity have been associated with efficient cognitive control and visuospatial processing, which in prior surgical studies have correlated with reduced task errors and shorter completion times [7, 8, 29, 33, 34]. Similarly, expert-like gaze patterns characterized by longer fixations and fewer saccades have been associated with optimized visual attention and smoother hand–eye coordination [9, 30].

### Strengths of this study

This study introduces deep learning methods for RAS scenarios to move beyond benchtop simulations. The inclusion of participants across varying experience levels and rigorous model validation on data from participants not used in model training ensures strong generalizability. The model’s ability to deliver precise, quantitative feedback supports its integration into training programs aimed at improving technical proficiency and consistency.

A key strength of this study is that the biometric-based assessment framework is independent of any specific robotic platform, which allows consistent evaluation across different systems and generations of RAS technology. Unlike metrics derived from system-integrated performance logs, which differ between manufacturers and even between generations of the same system, physiological and gaze-based indicators reflect intrinsic cognitive and psychomotor processes that underlie surgical performance, independent of hardware characteristics.

This distinction is practically meaningful, as recent studies demonstrated that postoperative outcomes can vary across robotic platforms. For example, lung resection procedures performed using the da Vinci Xi system were associated with lower postoperative complication rates compared to the earlier Si platform, which may reflect differences in system ergonomics, visualization, and instrumentation [35]. Our EEG– and eye-tracking–based skill assessment method provides a complementary pathway toward evaluating surgeon proficiency that does not rely on platform-specific technology that supports consistent training standards across institutions and robotic systems. Focus on surgeon neurophysiology and visual behavior rather than device-specific metrics lays the groundwork for more universal, transferable assessment and education models that can help ensure equitable, high-quality training regardless of the robotic platform employed.

### Limitations of this study and future work

Despite promising results, model generalizability to other surgical tasks is yet to be established. Although the sample size of 23 participants is relatively small for deep learning, the use of subject-independent splits and 10 repeated test runs provides a conservative and robust estimate of generalization performance. Future work should explore broader surgical contexts and develop automated task segmentation to streamline data processing. Expanding the rater pool beyond three raters will improve labeling reliability and reduce potential bias. Increasing the sample size could further improve reliability and generalizability of the findings.

Current limitations, such as manual video analysis, reliance on three raters, and lack of functional anastomosis outcomes measurements such as leak testing and post-operative complications, emphasize the need for further methodological refinement. Incorporating more expert raters and automated annotations will enhance robustness and facilitate broader adoption in surgical education.

Future work should explore integrating multimodal large language models (MLLMs) and vision–language models with physiological signals and surgical video to enable richer contextual understanding of instrument–tissue interaction, temporal task structure, and cognitive–visuomotor dynamics. Recent advances in surgical MLLMs demonstrate strong capability in capturing spatial and temporal information from operative video, and their integration with EEG and eye-tracking data may further enhance the granularity and generalizability of objective skill assessment [36].

## Supplementary Information

Below is the link to the electronic supplementary material.

Supplementary Material 1

## Data Availability

The datasets generated during and/or analyzed during the current study are available from the corresponding author (SBS) upon reasonable request.
