# Supplementary material for: Multimodal deep learning for objective skill assessment in robot-assisted vesico-urethral anastomosis
Source: J Robot Surg. 2026 Mar 10;20(1):337. doi: 10.1007/s11701-026-03290-z (PMC12971764; doi:10.1007/s11701-026-03290-z)
Supplement: Supplementary file 1 — Supplementary Material 1 [file 11701_2026_3290_MOESM1_ESM.docx]

**Supplement 1**

Domains of the Robotic Anastomosis Competency Evaluation metrics (RACE) tool.


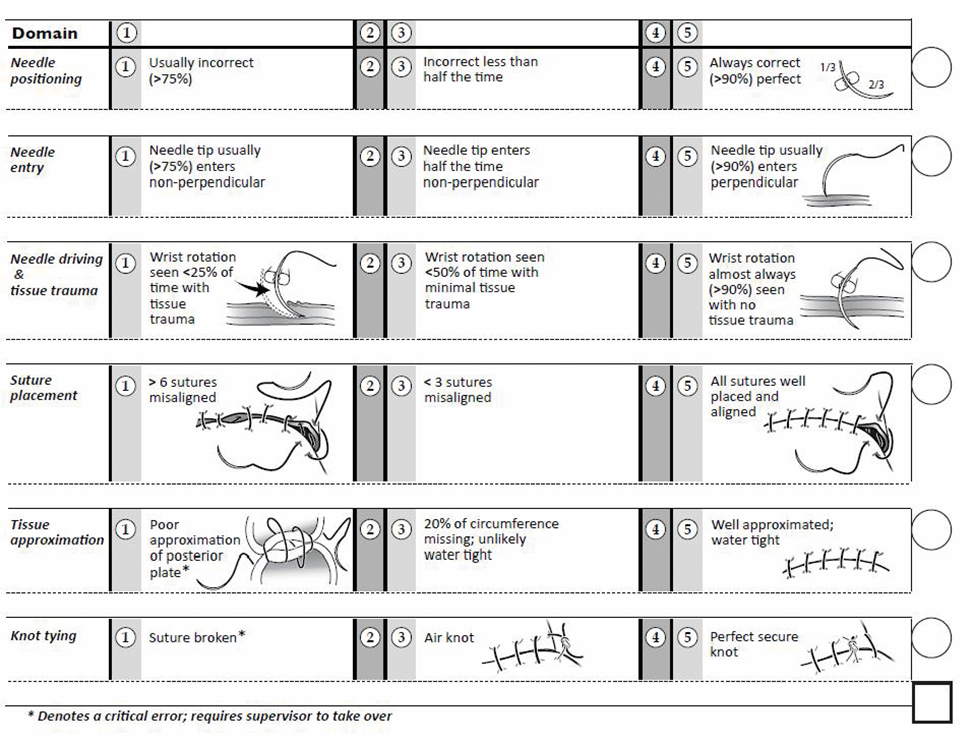


Figure 1 Robotic Anastomosis Competency Evaluation form.
